# Asparagine Availability Is a Critical Limiting Factor for Infectious Spleen and Kidney Necrosis Virus Replication

**DOI:** 10.3390/v16101540

**Published:** 2024-09-29

**Authors:** Baofu Ma, Fangying Li, Xiaozhe Fu, Xia Luo, Qiang Lin, Hongru Liang, Yinjie Niu, Ningqiu Li

**Affiliations:** Pearl River Fishery Research Institute, Chinese Academy of Fishery Sciences, Key Laboratory of Fishery Drug Development, Ministry of Agriculture and Rural Affairs, Guangdong Province Key Laboratory of Aquatic Animal Immune and Sustainable Aquaculture, Guangzhou 510380, China

**Keywords:** *Siniperca chuatsi*, ISKNV, asparagine, aspartate-malate shuttle, asparagine synthetase

## Abstract

Infectious spleen and kidney necrosis virus (ISKNV) has brought huge economic loss to the aquaculture industry. Through interfering with the viral replication and proliferation process that depends on host cells, its pathogenicity can be effectively reduced. In this study, we investigated the role of asparagine metabolites in ISKNV proliferation. The results showed that ISKNV infection up-regulated the expression of some key enzymes of the asparagine metabolic pathway in Chinese perch brain (CPB) cells. These key enzymes, including glutamic oxaloacetic transaminase 1/2 (GOT1/2) and malate dehydrogenase1/2 (MDH1/2) associated with the malate-aspartate shuttle (MAS) pathway and asparagine synthetase (ASNS) involved in the asparagine biosynthesis pathway, were up-regulated during ISKNV replication and release stages. In addition, results showed that the production of ISKNV was significantly reduced by inhibiting the MAS pathway or reducing the expression of ASNS by 1.3-fold and 0.6-fold, respectively, indicating that asparagine was a critical limiting metabolite for ISKNV protein synthesis. Furthermore, when asparagine was added to the medium without glutamine, ISKNV copy number was restored to 92% of that in the complete medium, indicating that ISKNV could be fully rescued from the absence of glutamine by supplementing asparagine. The above results indicated that asparagine was a critical factor in limiting the effective replication of ISKNV, which provided a new idea for the treatment of aquatic viral diseases.

## 1. Introduction

Infectious spleen and kidney necrosis virus (ISKNV) is a fish-pathogenic virus belonging to the genus Megalocytivirus of the family Iridoviridae and has a large double-stranded DNA-containing icosahedral structure [1,2]. ISKNV causes serious viral disease with high morbidity and mortality of freshwater and marine fishes [3]. In particular, ISKNV can cause spleen and kidney enlargement of *Siniperca chuatsi* with edible and economic value, and the fatality rate is 100% [4]. However, few effective measures were available for control of ISKNV disease. A virus is a kind of non-cellular organism that needs to manipulate the host’s metabolic machinery to facilitate viral infection and pathogenicity. An in-depth understanding of the mechanism of viral replication and proliferation is of great significance for controlling and preventing ISKNV disease.

The virus hijacks the host’s metabolic machinery to promote its own replication. Glucose is the major carbon source for cellular biosynthesis and energy generation for cellular function and biomacromolecule synthesis [5]. Some studies showed that ISKNV infection changed glucose metabolism and affected the expression of enzymes related to the pentose phosphate pathway and glycolysis pathway during glucose metabolism, thereby enhancing the replication and proliferation of ISKNV [6,7,8]. ISKNV infection also enhanced the metabolic pathway of the tricarboxylic acid (TCA) cycle at the early stage of the ISKNV infection cycle, possibly through affecting glycolysis [8]. Glutamine plays an important role in the production process of cells, providing intermediates and energy for the TCA cycle, and is a precursor of many non-essential amino acids [9,10]. Some studies showed that glutamine and glutamine-derived α-ketoglutarate (αKG) were necessary for the efficient replication and proliferation of ISKNV [11,12]. The production of ISKNV was significantly reduced in the culture medium without glutamine [12]. Supplementation of TCA cycle intermediates such as αKG, oxaloacetate, and citrate can promote the replication and proliferation of ISKNV. Among them, the production of ISKNV was the highest when citrate was supplemented [12]. In addition, ISKNV induced reductive glutamine metabolism in the host cells through the mTOR/PGC-1α/SIRT3 Pathway, thereby increasing the replication of ISKNV [11]. During the proliferation process, cells are more dependent on glutamine than other non-essential amino acids [13,14]. Glutamine is converted into glutamate by glutaminase and then converted into α-ketoglutarate by glutamate dehydrogenase to enter the tricarboxylic acid cycle, ultimately providing energy and a carbon source [15,16]. In addition, glutamine is involved in the conversion of aspartate to asparagine via asparagine synthetase (ASNS) in an ATP-dependent reaction [17]. Asparagine is an important amino acid and is widely used for the production of other biomolecules such as proteins, lipids, nucleotides, and glucose. Asparagine has been found to maintain viability of glutamine-deprived cells without the need for restoration of supplementation or levels of other non-essential amino acids [18]. Aspartate synthesis also plays an important role in the mitochondrial electron transport chain (ETC) during cell proliferation [19]. Furthermore, asparagine has also been reported to play a vital role in the development of cancer cells. Nutritional inhibition by targeting asparagine is often considered an anti-cancer strategy and has shown success in the treatment of cancer [20]. However, it is not clear about the relationship between ISKNV replication and asparagine metabolism.

**Scheme 1:**
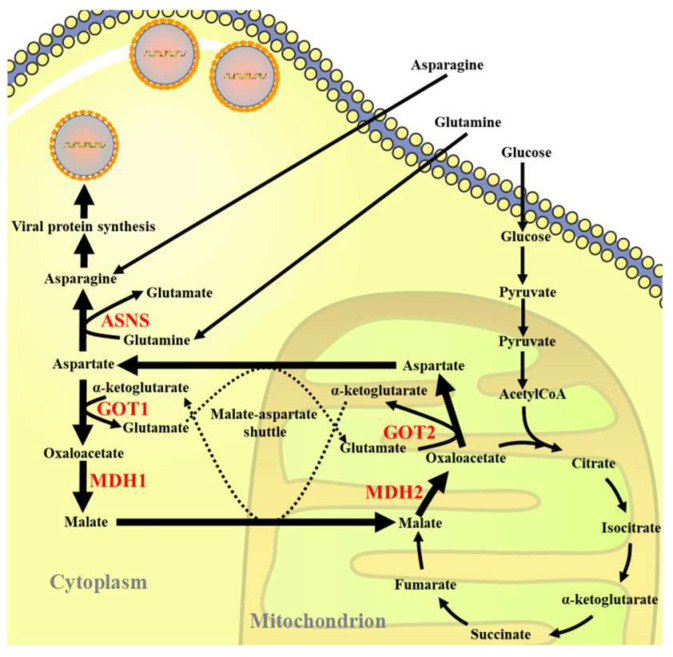
Schematic illustration of the role of glutamine in the efficient replication of ISKNV in CPB cells.

In this study, we investigated the role of asparagine metabolites in ISKNV proliferation. The results showed that ISKNV infection could upregulate the expression of some key enzymes of the asparagine metabolic pathway in Chinese perch brain (CPB) cells. Further studies showed that the production of ISKNV was significantly reduced by inhibiting the MAS pathway or reducing the expression of ASNS. Furthermore, ISKNV could be fully rescued from the absence of glutamine by supplementing asparagine (Scheme 1). The above results indicated that asparagine was a critical factor in limiting the effective replication of ISKNV, which provided a new idea for the treatment of aquatic viral diseases.

## 2. Materials and Methods

### 2.1. Cell Lines and Viral Strains

Chinese perch brain (CPB) cells were extracted and immortalized from the *Siniperca chuatsi* brain and stored in our laboratory [21]. The cells were cultured in L-15 medium (Servicebio, Wuhan, China) supplemented with 10% FBS at 28 °C in an incubator. The ISKNV strain was also isolated, identified, and preserved by our laboratory [22]. The ISKNV was cultured in an incubator using CPB cells, and its titer was determined by calculating TCID50 using the Reed-Muench method, which was 10^−7.8^. Viruses were stored at −80 °C until use.

### 2.2. Reagents and Antibodies

L-glutamine (Gln, Q), D-glucose (Glc), and L-asparagine (Asn, N) were purchased from Sigma-Aldrich (St. Louis, MO, USA). L-asparagine was dissolved in sterile deionized water (Solarbio, Beijing, China) by a 60 °C water bath. All the above chemicals were diluted to the specified final concentrations in the medium before use. The rabbit anti-ISKNV-MCP antibodies were developed and stored in our laboratory [23]. The rabbit anti-ASNS, MDH1/2, GOT1/2, and the mouse anti-β-actin monoclonal antibodies were obtained from Proteintech (Rosemont, IL, USA). Goat anti-mouse IgG and goat anti-rabbit IgG were obtained from Sigma (St. Louis, MO, USA). Aminooxyacetic Acid (AOAA) hemi-hydrochloride was obtained from MCE (Princeton, NJ, USA) and diluted to the specified final concentrations with PBS before use.

### 2.3. Cell Viability Assay

The cytotoxicity of glutamine depletion and rescue on CPB cells were assessed by the cell counting kit-8 (CCK-8). Briefly, CPB cells cultured in L-15 medium were seeded on 96-well plates and grown in an incubator. When the cells grew to approximately 90% confluence, they were washed twice with phosphate buffered saline (PBS) and then cultured with fresh glutamine-deficient medium containing 2 mM L-asparagine or 2 mM L-glutamine for 72 h, respectively. Subsequently, the CCK8 was added into wells and incubated for 4 h. The optical density was measured on a microplate reader (Infinite M200 Pro, Tecan, Männedorf, Swiss) at 450 nm. The wells with glutamine-free medium were defined as a control with 100% viability; the viabilities of other groups were calculated relative to the group.

### 2.4. Glutamine Depletion and Rescue

A special DMEM medium lacking L-glutamine, D-glucose, phenol red, and sodium pyruvate (Gibco, Grand Island, NY, USA) was used to study the effect of glutamine deficiency on ISKNV virus replication. Briefly, the medium was supplemented with 2 mM L-glutamine, 1 g/L D-glucose, 2 mM L-asparagine, and 2% dialyzed fetal bovine serum (Hyclone, Logan, UT, USA) for glutamine depletion rescue and experiments. All cells were cultured in the above DMEM medium at 28 °C and 5% CO_2_. The cells were washed with PBS before treatment with the above special DMEM.

### 2.5. Determination of TCID_50_ of ISKNV Virus

The intracellular (the cells containing viruses), supernatant (the cell culture medium containing viruses), and whole sample virus fluids (the cells and cell culture medium containing viruses) of the ISKNV infection group with or without the addition of the inhibitor AOAA hemi-hydrochloride were collected, and then the virus fluids were serially diluted in a centrifuge tube with L-15 medium, with a dilution range of 10^−1^ to 10^−10^. The diluted virus fluid was added to a 96-well plate, and each dilution gradient was repeated eight times, with 100 μL per well. Cells without any treatment were used as the control group, and the cell status was observed every day. The data were recorded for two consecutive weeks, and the results were calculated using the Reed-Muench two-way method.

### 2.6. The Transcriptional Expression of Genes Was Detected by qPCR

Total RNA from cells was extracted using a cell total RNA isolation kit (FOREGENE, Chengdu, China) and then reverse transcribed into cDNA according to the protocol of the manufacturer of TransScript^®^ All-in-One First-Strand cDNA Synthesis SuperMix for qPCR (One-Step gDNA Removal) (TransGen Biotech, Beijing, China). To obtain the cDNA core fragment of ASNS, GOT1/2, and MDH1/2, the primers used were listed in Table 1. These cDNAs were amplified on a real-time PCR instrument using the SYBR Green Pro Taq HS kit (Accurate Biology, Changsha, China). The reaction volume of SYBR Green was 20 μL, including 10 μL 2 × SYBR Premix Enzyme containing ROX, 1 μL each of forward and reverse primer (10 μM), and 6 μL ddH_2_O and 2 μL cDNA. All reactions were repeated three times.

### 2.7. DNA Extraction and Quantification of ISKNV Genome Copies

Viral DNA in the intracellular (the cells containing viruses) and supernatant (the cell culture medium containing viruses) of ISKNV-infected CPB cells was extracted using the Easy Pure Simple virus DNA/RNA kit (TransGen Biotech, Beijing, China). The used primers and probe were listed in Table 1. The reaction system contained 10 μL 2 × Premix, 0.5 μL primers (10 μM), 0.5 μL Probe, 0.5 μL ROX, and 6 μL ddH_2_O. The procedure was 94 °C for 1 min, 94 °C for 10 s, 60 °C for 30 s, 40 cycles. Each sample was repeated 3 times.

### 2.8. Western Blot

CPB cells were collected and then lysed with radio immunoprecipitation assay (RIPA) buffer containing 1 mM phenylmethanesulfonyl fluoride (PMSF). Proteins were denatured by boiling for 10 min and then loaded into sodium dodecyl sulfate-polyacrylamide gel electrophoresis for fractionation. Subsequently, the proteins on gel were then transferred onto a polyvinylidene difluoride (PVDF) membrane, and the membrane was blocked with 5% (*m*/*v*) skim milk powder for 3 h. The primary antibodies GOT1 (1:500), MDH1 (1:500), GOT2 (1:500), MDH2 (1:1000), ASNS (1:400), ISKNV-MCP (1:1000), and β-actin (1:3000) were diluted and then incubated with the membrane overnight at 4 °C. Subsequently, the membranes were incubated with HRP-conjugated secondary antibodies for 1 h at room temperature with shaking. Finally, the membranes were visualized by the chemiluminescent substrate.

### 2.9. RNA Interference

The small interfering RNA (siRNA) sequence of ASNS was obtained from Shanghai GenePharma Co., Ltd. (Shanghai, China) (Table 2). TransIntro^®^ EL transfection reagent (Trans-Gen Biotech, Beijing, China) was used to transfect negative control (NC) and si-ASNS into CPB cells at a density of 70% to 80%, and then the cells were collected 24 h and 72 h after transfection, respectively. The optimal siRNA sequence for knocking down ASNS was determined by qPCR and Western blot. Subsequently, NC and the siRNA with the best knockdown effect were transfected into CPB cells for 4 h, and then ISKNV was inoculated into the cells for 72 h. Finally, cells were collected for qPCR and Western blot, and then the effect of ASNS knockdown on ISKNV viral DNA and protein was analyzed.

### 2.10. Statistical Analysis

All data were obtained from three parallel experiments and analyzed by one-way or two-way ANOVA. All data compared with the control group were significantly annotated as follows: NS, *p* > 0.05, * *p* < 0.05, ** *p* < 0.01, *** *p* < 0.001.

## 3. Results

### 3.1. ISKNV Infection Altered Asparagine Metabolism

**Figure 1:**
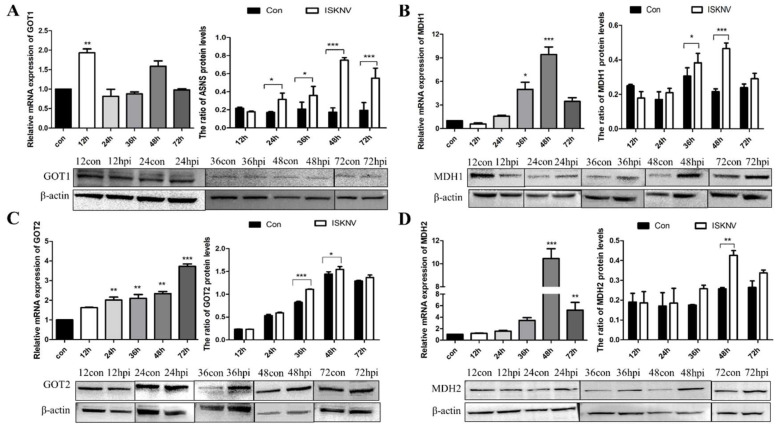
ISKNV infection altered the MAS pathway in CPB cells. Transcript and protein levels of the key enzyme genes GOT1 (**A**), MDH1 (**B**), GOT2 (**C**), and MDH2 (**D**) in the MAS pathway, respectively. And hpi, hour post infection. * *p* < 0.05, ** *p* < 0.01, *** *p* < 0.001 (unpaired Student’s *t*-test); data are representative of three different independent experiments (mean ± SEM).

**Figure 2:**
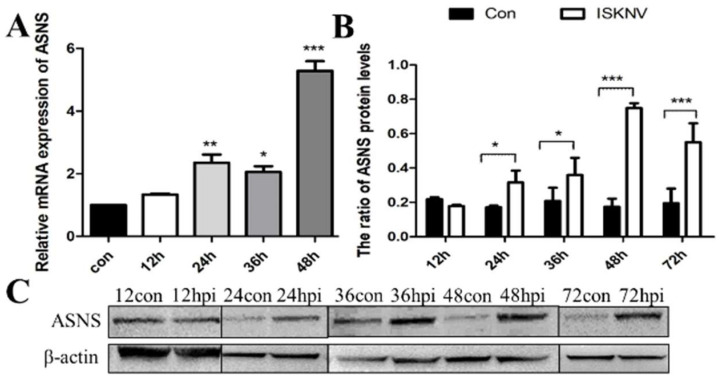
ISKNV infection altered the asparagine biosynthesis pathway in CPB cells. Transcript (**A**) and protein (**B**,**C**) levels of the key enzyme gene ASNS in the asparagine biosynthesis pathway, respectively. And hpi, hour post infection; * *p* < 0.05, ** *p* < 0.01, *** *p* < 0.001 (unpaired Student’s *t*-test); data are representative of three different independent experiments (mean ± SEM).

Asparagine is critical for coordinating cellular responses to amino acid homeostasis, overall protein synthesis, and metabolic availability during biological processes and disease development [25]. To investigate the role of asparagine metabolism in ISKNV proliferation, expression of key enzymes in the asparagine metabolic pathway (GOT1, MDH1, GOT2, MDH2, and ASNS) in the mRNA and protein levels was determined by qPCR and Western blot in CPB cells at different time points. As shown in Figure 1A, the mRNA levels of GOT1, a key enzyme in the malate-aspartate shuttle (MAS) pathway, increased significantly at 12 hpi. Similarly, three other key enzymes of the MAS pathway also changed. The expression of MDH1 was significantly up-regulated from 36 to 48 hpi (Figure 1B), and the expression of GOT2 was also significantly up-regulated from 36 to 48 hpi (Figure 1C). The mRNA and protein levels of MDH2 increased significantly at 48 hpi (Figure 1D). In addition, the expression of ASNS, a key enzyme in the asparagine biosynthesis pathway, was significantly up-regulated from 24 to 72 hpi (Figure 2). These results indicated that ISKNV mainly promoted the expression of GOT1, MDH1, GOT2, and MDH2 in the early stage of infection and further up-regulated the expression of ASNS in the late stage of infection. ISKNV infection could promote the MAS pathway in the early stage of replication to facilitate energy supply and increase the asparagine biosynthesis pathway in the late stage of replication to promote the synthesis of viral proteins.

### 3.2. Inhibition of the MAS-Affected ISKNV Replication

**Figure 3:**
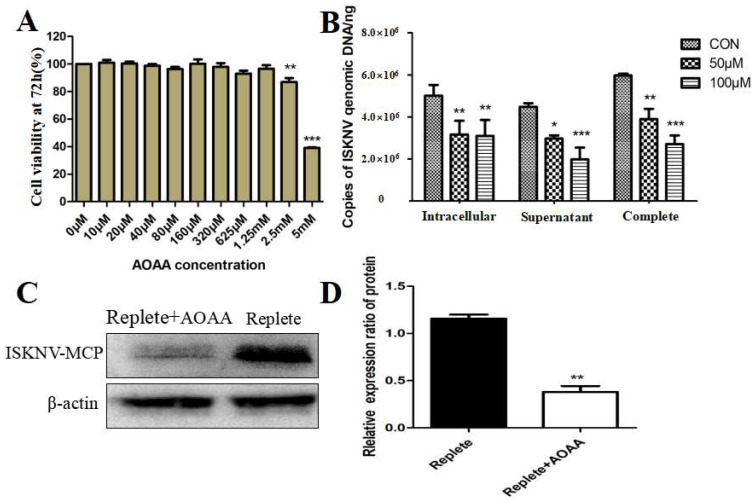
Inhibition of the MAS pathway affects ISKNV replication. (**A**) Cell viability of CPB cells treated with different concentrations of an AOAA for 72 h. (**B**) Effect of adding AOAA inhibitor on ISKNV virus production. (**C**,**D**) Effect of adding inhibitor AOAA on the expression of ISKNV viral proteins. And * *p* < 0.05, ** *p* < 0.01, *** *p* < 0.001 (unpaired Student’s *t*-test); data are representative of three different independent experiments (mean ± SEM).

The MAS comprises a transport–transamination–redox cycle in both cytosolic and mitochondrial domains [26]. Previous studies have shown that MAS plays an important role in a variety of biological processes, such as the synthesis of glutamate and glutamine in cells [27,28]. Aminooxyacetic acid (AOAA) is a widely used MAS inhibitor that can lead to reduced intracellular ATP levels, changes in cell cycle, glycolysis rate, extracellular lactate, and pyruvate levels by inhibiting glycolysis [29]. To further confirm whether ISKNV replication was related to the activation of the MAS pathway, we used AOAA to investigate the effect of MAS on ISKNV replication. Cell viability was first used to characterize the cytotoxicity of AOAA. As shown in Figure 3A, AOAA with 0–625 μM had no significant effect on the viability of CPB cells. Subsequently, the addition of 50 and 100 μM AOAA to complete medium reduced viral titers in ISKNV-infected cells, supernatants, and whole samples (Table 3). In addition, the effect of AOAA on ISKNV production was assessed by qPCR. As shown in Figure 3B, the production of ISKNV was significantly reduced within the safe concentration range. Furthermore, Western blot was used to further assess the effect of AOAA on ISKNV protein expression. As shown in Figure 3C,D, the protein expression of ISKNV-MCP was significantly down-regulated. All of the above results suggested that MAS inhibitors could reduce the replication and proliferation of ISKNV.

### 3.3. ASNS Knockdown Reduced ISKNV Replication

**Figure 4:**
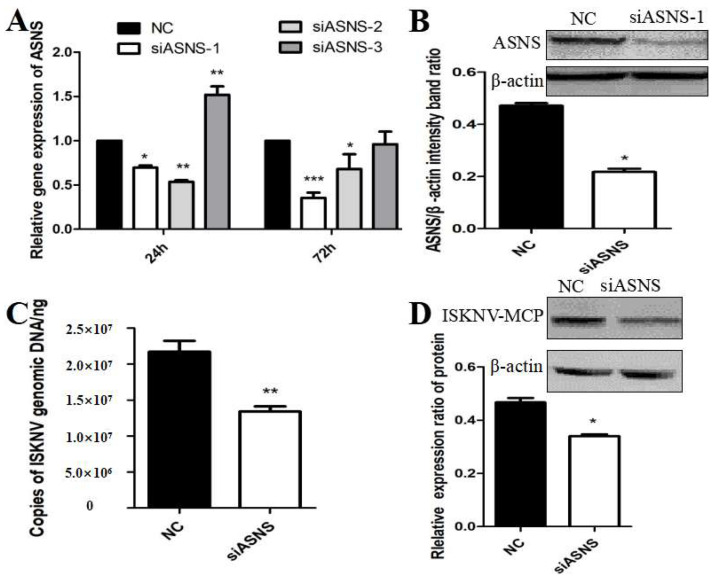
Effects of ASNS knockdown on ISKNV replication. (**A**,**B**) Effects of siASNS treatment on ASNS mRNA and protein expression in CPB cells. (**C**) Effects of siASNS treatment on ISKNV copy number in CPB cells. (**D**) Effects of siASNS treatment on ISKNV-MCP protein expression in CPB cells. And * *p* < 0.05, ** *p* < 0.01, *** *p* < 0.001 (unpaired Student’s *t*-test); data are representative of three different independent experiments (mean ± SEM).

The nonessential amino acid asparagine can only be synthesized de novo by the enzymatic activity of asparagine synthetase (ASNS) [30]. To further confirm whether ISKNV replication was related to the activation of the biosynthetic pathway of asparagine, we designed and synthesized siRNAs that interfered with ASNS. As shown in Figure 4A,B, after siASNS-1 treatment of cells for 72 h, the mRNA and protein levels of ASNS were significantly decreased compared with the NC group, indicating that siASNS-1 could knock down the expression of ASNS. To further evaluate the effect of ASNS knockdown on ISKNV replication, CPB cells were treated with si-ASNS and then infected with ISKNV for 24 h. The results suggested that the copy number of ISKNV in CPB cells was significantly reduced after ASNS knockdown (Figure 4C). In addition, ISKNV-MCP protein synthesis was also down-regulated in ASNS siRNA-treated cells (Figure 4D). These results indicated that asparagine biosynthesis played an important role in ISKNV replication.

### 3.4. Asparagine Fully Rescued ISKNV Replication in Glutamine Depletion

**Figure 5:**
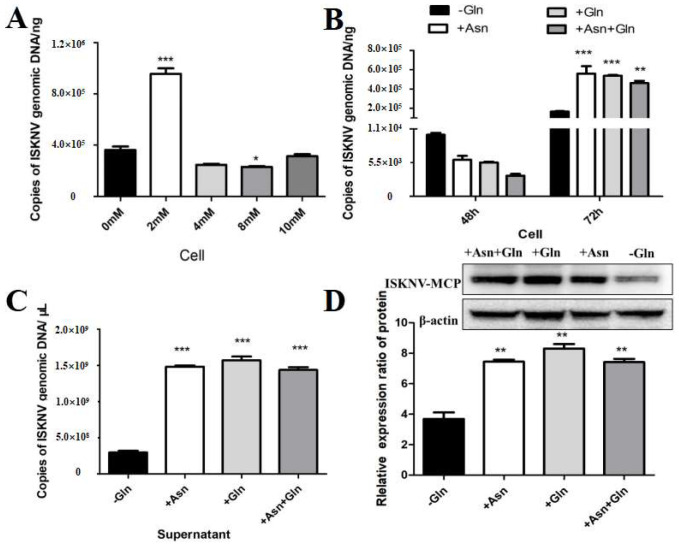
Asparagine recused ISKNV replication in glutamine depletion. (**A**) The copy number of ISKNV with different concentrations of asparagine. (**B**,**C**) The copy number of ISKNV in medium supplemented with 2 mM asparagine (Asn, N) and 2 mM glutamine (Gln, Q). (**D**) The expression levels of ISKNV-MCP protein in CPB cells in medium supplemented with 2 mM asparagine (Asn, N) medium. And * *p* < 0.05, ** *p* < 0.01, *** *p* < 0.001 (unpaired Student’s *t*-test); data are representative of three different independent experiments (mean ± SEM).

Encouraged by the above results, we further evaluated whether asparagine could fully rescue ISKNV replication under conditions of glutamine depletion. In our experiment, the viral yield at different asparagine addition concentrations was assessed by qPCR to confirm the optimal asparagine addition concentration for rescuing ISKNV replication. As shown in Figure 5A, the addition of 2 mM asparagine significantly increased ISKNV replication. To further evaluate the effect of asparagine on viral production during the ISKNV replication cycle, we analyzed the effect of asparagine supplementation on viral DNA and protein synthesis in CPB cells grown in glutamine-depleted cultures by qPCR and Western blot. The results showed that glutamine depletion significantly reduced viral production, but ISKNV production was fully restored when asparagine and glutamine were added to the medium 72 h after ISKNV infection (Figure 5B,C). The above results indicated that glutamine and asparagine are required for the late stage of ISKNV proliferation. Notably, the addition of asparagine did not improve viral production of ISKNV when glutamine was present in the medium, suggesting that glutamine or asparagine can similarly rescue viral replication of ISKNV. In addition, we assessed the expression of the ISKNV-MCP protein by Western blot. As shown in Figure 5C, the addition of asparagine to glutamine-deficient medium promoted MCP protein synthesis in ISKNV-infected cells, indicating that asparagine can rescue MCP protein synthesis in glutamine-deficient cells. Taken together, these results showed that asparagine could fully restore ISKNV production in the absence of glutamine.

## 4. Discussion

Viruses do not have their own metabolic mechanisms, and their replication depends on the host to provide nutrients and energy. Many viruses can induce changes in the metabolic pathways of host cells, thereby enhancing their proliferation and ultimately aggravating the degree of viral infection [31,32]. Therefore, it is helpful to explore the mechanism of viral proliferation to aid in treating viral diseases. This study evaluated whether asparagine is the key limiting amino acid for ISKNV replication by investigating key enzymes of the MAS pathway and the asparagine biosynthesis pathway, which would also explain the dependence of ISKNV replication on glutamine [11].

The MAS comprises a transport–transamination–redox cycle in both cytosolic and mitochondrial domains [26]. Mitochondria use the MAS (also known as the malate shuttle) to transport electrons produced during glycolysis to the mitochondrial impermeable inner membrane for oxidative phosphorylation, which allows the hydrogen ions of the cofactor NADH produced in the cell cytosol to reach the electron transport chain in the mitochondria and ultimately produce ATP [33,34]. Previous studies have shown that MAS plays an important role in a variety of biological processes, such as the synthesis of glutamate and glutamine in cells [27,28]. Herein, we investigated the role of the MAS pathway in ISKNV proliferation, and the results showed that ISKNV could alter the MAS pathway in CPB cells. ISKNV mainly promoted the expression of MDH1, GOT1, MDH2 and GOT2 in the early stage of infection and further facilitated energy supply, ultimately promoting the proliferation of ISKNV. A previous study has shown that fetal liver hematopoietic stem cells (FL-HSCs) have enhanced mitochondrial respiration and a glycolytic level similar to maintain their stemness through activation of the MDH1-mediated malate-aspartate NADH shuttle [35]. GOT activity was also elevated in some virus-infected cells, which may lead to a reversible transition between the TCA cycle and amino acid synthesis [36]. Similarity: our previous studies have shown that *Siniperca chuatsi* rhabdovirus (SCRV) infection promoted the expression of key enzymes in the MAS pathway in the early stage, driving the MAS [24]. AOAA is a widely used MAS inhibitor and used to study the role of the MAS pathway [29]. To further confirm whether ISKNV replication was related to the activation of the MAS pathway, we used AOAA to investigate the effect of MAS on ISKNV replication. The results suggested that MAS inhibitors could reduce the replication and proliferation of ISKNV, which implied that the MAS pathway was an indispensable part of ISKNV proliferation.

The nonessential amino acid asparagine can only be synthesized de novo by the enzymatic activity of asparagine synthetase (ASNS) [30]. We investigated the role of the asparagine biosynthesis pathway in ISKNV proliferation, and the results showed that the mRNA and protein levels of ASNS were significantly up-regulated in ISKNV-infected cells at the late stage of viral replication, indicating that ISKNV infection promoted the synthesis of asparagine from aspartate. To further confirm whether ISKNV replication was related to the activation of the biosynthetic pathway of asparagine, we designed and synthesized siRNAs that interfered with ASNS. Our results found that ISKNV replication was significantly inhibited when ASNS was knocked down in the presence of glutamine. ASNS play an important role in the replication and proliferation of human cytomegalovirus (HCMV) and vaccinia virus (VACA) [37,38]. Furthermore, adenovirus infection up-regulated the expression of ASNS in infected cells [39]. Encouraged by the above results, we further evaluated whether asparagine could fully rescue ISKNV replication under conditions of glutamine depletion. These results showed that asparagine could fully restore ISKNV production in the absence of glutamine, suggesting that asparagine availability was a critical limiting factor for efficient replication of infectious spleen and kidney necrosis virus in Chinese perch brain cells. In addition, some studies have shown that glutamine and glutamine-derived α-ketoglutarate (αKG) were necessary for the efficient replication and proliferation of ISKNV [11,12]. Since de novo synthesis of asparagine requires the use of glutamine as an amino donor, asparagine cannot be fully synthesized without exogenous glutamine [40]. The above results suggest that ISKNV needs glutamate to synthesize aspartate and ultimately synthesize viral proteins.

Based on the above results, we hypothesize that the replication of ISKNV in fish can be inhibited by limiting the supply of glutamate or inhibiting the key enzyme activities of the MAS pathway and the asparagine biosynthesis pathway. In detail, these methods include adjusting the feed formula, using some chemical inhibitors, or using knockdown expression technology to inhibit the replication of ISKNV. Therefore, the antiviral capacity of fish is enhanced by inhibiting the synthesis of asparagine, which may become a new antiviral method.

## 5. Conclusions

In summary, ISKNV infection hijacked multiple key enzymes to accelerate the aspartate metabolic pathway in CPB cells to provide energy and biomolecules for viral replication and proliferation. The MAS pathway was required for ISKNV replication and proliferation. In addition, ISKNV replication was highly dependent on asparagine levels, and inhibition of key enzymes involved in asparagine synthesis leads to severe attenuation of viral replication. These findings suggested that key enzymes of the asparagine metabolic pathway may be new targets for effective control of ISKNV infection and provide new ideas for therapeutic strategies for ISKNV disease.

## Figures and Tables

**Table 1:** Quantitative real-time polymerase chain reaction primers [24].

Primer Name	Gene Name	Sequence (5′-3′)
ASNS-F	asparagine synthetase	GAAATGTGTGGTATCTGGGCTTTGT
ASNS-R		TCAAAGACTTCAAAGTGGCCAGGG
MDH2-F	malate dehydrogenase 2	ACTGCCCCGAAGCTATGA
MDH2-R		CAGCGTGACCTCCAATGAC
GOT2-F	Glutamic-oxaloacetic transaminase	ACCATGGCCCTGCTCAAG
GOT2-R		TACTTGGTGACAGCATGGATCGC
GOT1-F	Glutamic-oxaloacetic transaminase 1	CGGCTTATCAGGGTTTCGC
GOT1-R		CAGGGCTGTTGAGGGTCTTG
MDH1-F	malate dehydrogenase 1	ATGGGTGTTTACTCCTCTGGC
MDH1-R		TTTGGGGCGGTCTAAGTG
18S-F	18S RNA	CATTCGTATTGTGCCGCTAGA
18S-R		CAAATGCTTTCGCTTTGGTC
ISKNV-F	ISKNV-007	CGAGGCCACATCCAACATC
SKNV-R		CGCCTTTAACGTGGGATATATTG
ISKNV-Probe		CACCAAACTGACCGCGGACTCGT

**Table 2:** The siRNA sequence of ASNS.

Name	Sequence (5′-3′)	Anti-Sequence (5′-3′)
siRNA-ASNS (siASNS-1)	GGCUUUGUUUGGCAGUGAUTT	AUCACUGCCAAACAAAGCCTT
siRNA-ASNS (siASNS-2)	GCAAGGUUGCAGCUCACAUTT	AUGUGAGCUGCAACCUUGCTT
iRNA-ASNS (siASNS-3)	GGUAUGUACUUGGUUUCAATT	UUGAAACCAAGUACAUACCTT
Negative control (NC)	UUCUCCGAACGUGUCACGUTT	ACGUGACACGUUCGGAGAATT

**Table 3:** ISKNV titers after treatment with different concentrations of inhibitor AOAA.

	Sample	Intracellular (TCID_50_/0.1 mL)	Supernatant (TCID_50_/0.1 mL)	Complete (TCID_50_/0.1 mL)
AOOA Concentration				
control		10^−4.23^	10^−4.16^	10^−4.71^
50 μM		10^−3.78^	10^−3.71^	10^−4.33^
100 μM		10^−3.29^	10^−3.13^	10^−3.6^

## Data Availability

The data that support the findings of this study are available from the corresponding author upon reasonable request.
